# Biological Performance of Titanium Surfaces with Different Hydrophilic and Nanotopographical Features

**DOI:** 10.3390/ma16237307

**Published:** 2023-11-24

**Authors:** Barbara Illing, Leila Mohammadnejad, Antonia Theurer, Jacob Schultheiss, Evi Kimmerle-Mueller, Frank Rupp, Stefanie Krajewski

**Affiliations:** Department Medical Materials Science & Technology, University Hospital Tübingen, Osianderstr. 2-8, 72076 Tübingen, Germany; barbara.illing@med.uni-tuebingen.de (B.I.); jacob.schultheiss@med.uni-tuebingen.de (J.S.); evi.kimmerle-mueller@med.uni-tuebingen.de (E.K.-M.);

**Keywords:** dental-implant interface, titanium, hydrophile, nanostructure, osseointegration, human gingival fibroblasts, plasma etching, R1234yF

## Abstract

The micro- and nanostructures, chemical composition, and wettability of titanium surfaces are essential for dental implants’ osseointegration. Combining hydrophilicity and nanostructure has been shown to improve the cell response and to shorten the healing time. This study aimed to investigate the biological response to different wettability levels and nanotopographical modifications in aged and non-aged titanium surfaces. By plasma etching titanium surfaces with the fluorine gas 2,3,3,3-tetrafluoropropene (R1234yF), additional nanostructures were created on the sample surfaces. Furthermore, this treatment resulted in sustained superhydrophilicity and fluoride accumulation. We examined the effect of various nanostructuring processes and aging using scanning electron microscopy, roughness analyses, and wettability measurement. In addition, all the surface modifications were tested for their effects on fibroblast adhesion, proliferation, and viability as well as osteoblast differentiation. Our study indicates that the plasma etching, with 2,3,3,3-tetrafluoropropene, of the machined and SLA surface neither favored nor had an adverse effect on the biological response of the SAOS-2 osteoblast cell line. Although the fluorine-plasma-etched surfaces demonstrated improved fibroblast cell viability, they did not lead to improved early osseointegration. It is still unclear which surface properties mainly influence fibroblast and osteoblast adhesion. Further physiochemical aspects, such as electrostatic interaction and surface tension, are crucial to be analyzed along with wettability and roughness.

## 1. Introduction

The insertion of bone-anchored titanium implants is, nowadays, the treatment of choice for patients suffering from tooth loss [1,2]. To guarantee long-term survival and functionality, two biological responses to the implant are essential. The first is proper osseointegration to provide a structural and functional connection between the bone tissue and the implant surface without the interference of soft-tissue cells [3,4]. The second process is the formation of a tight soft-tissue seal created by gingival cells, such as fibroblasts and oral keratinocytes, around the implants’ neck to provide protection against invading pathogens and bacteria [5,6,7]. Among others, hydrophilicity is described to influence the biological response to the implant and, hence, may play a crucial role in the performance and success of dental implants [8]. Hydrophilicity can improve osseointegration; the process of implant integration into the surrounding bone tissue; as well as the adhesion and proliferation of gingival cells, such as fibroblasts and oral keratinocytes [9]. Hydrophilic surfaces promote better wetting and fluid transport at the implant–tissue interface, thereby enhancing protein adsorption from surrounding saliva and blood and resulting in improved clot formation on the implant surface. In addition, surface wettability affects the quantity and binding strength of bound proteins, their conformation and orientation, as well as the composition of the macromolecular films formed on them. Adsorbed proteins interact with cell-membrane receptors and activate biological pathways. The expression of the receptors on cells’ surfaces varies depending on their type and differentiation stage. Additionally, these receptors control short- and long-term processes, such as proliferation and differentiation as well as initial cellular attachment [10]. Furthermore, hydrophilic implant surfaces with high wettability form a stable and uniform liquid film when in contact with biological fluids, which can improve cell proliferation and differentiation [11,12]. Hence, the hydrophilic surface properties of dental-implant materials can significantly impact their long-term performance and success rate.

The second implant surface characteristic that plays a crucial role in long-term success is the micro- and nanostructures. Nano- and microstructured implant surfaces have a larger surface area compared to smooth ones, which can increase the availability of protein-based recognition sequences for cell integrin binding, thereby promoting cell adhesion [13,14,15]. The size, shape, and distribution of these structures can also provide a topographical cue for cells to align and differentiate along the direction of the surface structures, further promoting cell adhesion, proliferation, and differentiation [16,17]. For example, studies have shown that micro- and nanoscaled topographical cues on the implant surface can enhance osteoblast adhesion and proliferation by providing them with a favorable microenvironment for cell attachment, growth, and function [18]. Similarly, nanostructured surfaces have been shown to promote the better adhesion and proliferation of fibroblasts, the key cells involved in the survival and function of the gingival tissue surrounding and sealing dental implants [19].

Various techniques have been developed to modify the surface properties of implant materials, like titanium, and to introduce micro- and nanostructures to them. The most common method is blasting, which refers to a procedure in which abrasive materials, like sand, glass, or aluminum oxide, are used to achieve a rough surface texture on an implant [20,21]. Blasting can be combined with acid etching, which involves immersing the implant in an acidic solution to dissolve the surface material and to create further pores and grooves [22,23]. These procedures increase the surface area and create surface features that improve cell adhesion and promote tissue integration. Moreover, plasma etching can efficiently generate nanostructured surfaces [24]. Using fluorinated gases, such as carbon tetrafluoride (CF4), the surfaces of polymers, like polystyrene, or metals, like aluminum, can be modified to improve cellular responses [25,26]. A study targeting plasma-etched titanium revealed the formation of a two-tier hierarchical topography, which supports cell growth and osteogenic differentiation [27]. Overall, providing cells with favorable micro- and nanostructures on the surface of dental-implant materials can play a decisive role in regulating cell behavior, promoting osseointegration and gingival health, and ultimately enhancing the stability and longevity of dental implants.

These findings so far suggest that combined hydrophilic surfaces and nanostructures in dental implants exert synergistic effects on cell performance. This may result in the better cell adhesion and proliferation of soft-tissue cells and osteoblasts than that with either feature alone. Therefore, the present study aimed to assess the effect of different nanotopographical features and varied wettability in non-aged and aged titanium surfaces on their biological performance in vitro. In detail, machined titanium discs were used as an original substrate, and the effects of different nanostructuring processes and aging were investigated using scanning electron microscopy as well as roughness and wettability measurements. All the surfaces were further evaluated in terms of the adhesion, proliferation, and viability of fibroblasts as well as osteoblast differentiation.

## 2. Materials and Methods

### 2.1. Surface Modifications

Machined grade 2 titanium (Ti) discs (15 mm in diameter and 1 mm in thickness; Institute Straumann AG, Basel, Switzerland) was used as the basic material. In addition to the machined reference samples (M), four different groups, which were divided due to their processing, were included in this study (Table 1). The SLA titanium discs, with their coarsely grit-blasted and acid-etched surfaces, as well as an M_nano_ titanium surface, which was generated by plasma cleaning machined titanium, treating it with sodium chloride (saline) in a hydrothermal process, and storing it in saline for several weeks, were also supplied by Institute Straumann AG. The treatment process of the M_nano_ discs resulted in the development of small nanodots on the smooth machined surface. Moreover, the M and SLA discs underwent plasma etching with 2,3,3,3-tetrafluoropropene (Diener electronic GmbH + Co. KG, Ebhausen, Germany). For plasma etching of the R1234yF-modified surfaces, 20% R1234yF and 80% O_2_ gas were used (generator frequency, MHz; RF power, 100 W; gas pressure, 0.4 mbar) for 15 min.

### 2.2. Pre-Treatment of Samples

To exclude contaminations caused by different manufacturing processes and ambient conditions during manufacturing and storage of the samples, all samples were cleaned for 20 min in oxygen plasma (100% O_2_; generator frequency, 40 kHz; RF power, 80 W; gas pressure of approximately 0.3 mbar) in a plasma chamber (DENTAPLAS PC, Diener electronic GmbH, Ebhausen, Germany) before the respective biological experiments. To further simulate aging and, thus, recontamination and re-hydrophobization, the samples were stored directly after cleaning in glass petri dishes under ambient conditions for 14 days before experiments started (referred to as “aged”). Sample discs, which were used directly after cleaning, are called “new”.

### 2.3. Surface Characterization

#### 2.3.1. Scanning Electron Microscopy (SEM) of the Different Surfaces

Nanotopographical surface features of each sample were characterized via field emission scanning electron microscopy (JSM-6500F, Jeol, Tokyo, Japan).

To assess the morphology and location of the cells on the test surfaces, the samples were fixed in 2% (*v*/*v*) glutaraldehyde overnight followed by ascending ethanol dehydration. Subsequently, the samples were critically point-dried. Samples were sputtered with Au-Pd and were characterized via scanning electron microscopy (LEO 1430, Zeiss, Oberkochen, Germany).

The chemical composition of the researched surfaces was detected via energy-dispersive X-ray spectroscopy (EDX) (EDX-ZKK-31 Detector, Röntec/Bruker, Berlin, Germany).

#### 2.3.2. Roughness

The topography of the different surface modifications was determined via confocal microscopy (MarSurf CM Explorer, Mahr GmbH, Göttingen, Germany). For this purpose, five discs for each surface modification were evaluated. On each disc, six different areas of 800 × 800 µm were measured with a 20× objective (0.6 numeric aperture). Roughness was analyzed using MountainsMap Imaging Topography Software (Version 9.1.9957, Digital Surf, Besançon, France). First, the surface was leveled using the least square plane leveling method and then an S-Filter (λs): Gauss of 300:1 (800 µm) was applied to remove noise. Additionally, an L-Filter (λc): Gauss of 0.05 mm was used to remove possible waviness. Arithmetic mean roughness heights (Sa) were calculated for each surface modification and were statistically analyzed.

#### 2.3.3. Surface Wettability

Hydrophilicity was quantified by measuring static water contact angle with a high-resolution drop shape analysis system (OCA 200, DataPhysics Instruments GmbH, Filderstadt, Germany). A 1 μL drop of ultrapure water (Milli-Q; Merck Millipore, Darmstadt, Germany) was automatically placed on the sample disc surface and was video-recorded at 25 frames/s during an evaluation period of 30 s. The apparent contact angle at the equilibrated state (at 10 s) was chosen to characterize the hydrophilicity of the surface. Contact angles were measured immediately after oxygen plasma cleaning (0 h) and 1 h, 2 h, 3 h, 1 d, 2 d, 3 d, 7 d, and 14 d after cleaning. In total, 5 samples per surface modification and timepoint were measured. In this study, we define contact angles between 0° and 10° as superhydrophilic, contact angles of 10° to 30° as strong hydrophilic, contact angles of 30° to 60° as moderately hydrophilic, and contact angles of 60° to 90° as low hydrophilic. Contact angles between 90° and 180° indicate increasingly worse wetting conditions.

#### 2.3.4. Biological Tests

In order to screen for biofunctionality and to test the suitability of the modified surfaces for the specific requirements of the different implantation sites, interactions with primary human gingival fibroblasts (HFG) and a human osteoblast-like cell line (SAOS-2) were investigated.

#### 2.3.5. Cultivation of Cells

Human gingival fibroblasts (HGF) (HFIB-G, Cat.-No. 121 0412, Provitro AG, Berlin, Germany) cultured at 37 °C and 5% CO_2_ in fibroblast growth medium (Provitro), containing 14% fetal calf serum and 1% antibiotics as supplements, were used between passages three and eight. Human primary osteogenic sarcoma (SAOS-2) cell lines (DSMZ GmbH, Braunschweig, Germany) were cultured at 37 °C and 5% CO_2_ in McCoy’s 5A medium (Sigma-Aldrich Chemie GmbH, Steinheim, Germany), containing 15% fetal bovine serum (Thermo Fisher Scientific, Darmstadt, Germany), 1% L-glutamine (Thermo Fisher Scientific, Darmstadt, Germany), and 1% penicillin and streptomycin (Thermo Fisher Scientific, Darmstadt, Germany).

#### 2.3.6. Adhesion, Viability, and Proliferation Assay

Initial cell adhesion of HGF cells was determined 1 h after seeding using Alamar blue dye (Fisher Scientific, Schwerte, Germany) and was measured via UV photometry (Berthold Technologies, Bad Wildbad, Germany) (according to the manufacturer’s instructions). Before staining, non-adhering cells were removed via rinsing. The proliferation of HGF cells on the different surface modifications was determined using the cell counting kit-8 (CCK-8) assay (Dojindo Laboratories, Kumamoto, Japan). After 10,000 cells/cm^2^ were pre-incubated for 24 h on the different specimens, the CCK-8 solution was added according to the manufacturer’s instructions, and the spectral absorbance was measured at 450 nm. This process was repeated 48 h and 72 h after the cells were seeded on the sample surfaces. Afterwards, crystal violet (Sigma-Aldrich, Taufkirchen, Germany) staining was performed and was macroscopically analyzed. To quantify the cell coverage rate, the dye eluate was measured photometrically at 550 nm (Tecan, Crailsheim, Germany). For each experiment, three independent experiments with four samples per surface modification, respectively, were carried out.

#### 2.3.7. Differentiation of SAOS-2

To simulate bone healing, osteoblasts at a density of 10,000 SAOS-2 cells/cm^2^ were seeded onto the different test surfaces and were incubated for 24 h to allow adhesion. The differentiation process was initiated by adding vitamin C, b-glycerophosphate, and dexamethasone (10 µL each per ml of medium) (Sigma-Aldrich, Taufkirchen Germany) After 7 d, 14 d, and 21 d, osteogenesis (mineralized deposits) of the osteoblasts was determined using alizarin red (Sigma-Aldrich, Taufkirchen, Germany) staining as described before [28]. The eluate was quantified via photometric measurement at 405 nm. (Tecan, Crailsheim, Germany) Each experiment was performed independently three times with three samples per surface modification.

#### 2.3.8. Statistical Analysis

Unless otherwise specified, data are represented as mean ± standard deviation (SD). Student’s *t* test was used to compare the means between two groups. Statistically significant differences between the means of three or more groups were determined using one-way analysis of variance (ANOVA). Afterwards, comparison of machined control groups with all other groups, corrected via post hoc Tukey’s test, was performed. All statistical analyses were performed with the statistical software package GraphPad Prism (version 9.4.1, GraphPad Software, San Diego, CA, USA). Statistical significance was defined as *p* < 0.05.

## 3. Results

### 3.1. Surface Characteristics

**Figure 1:**
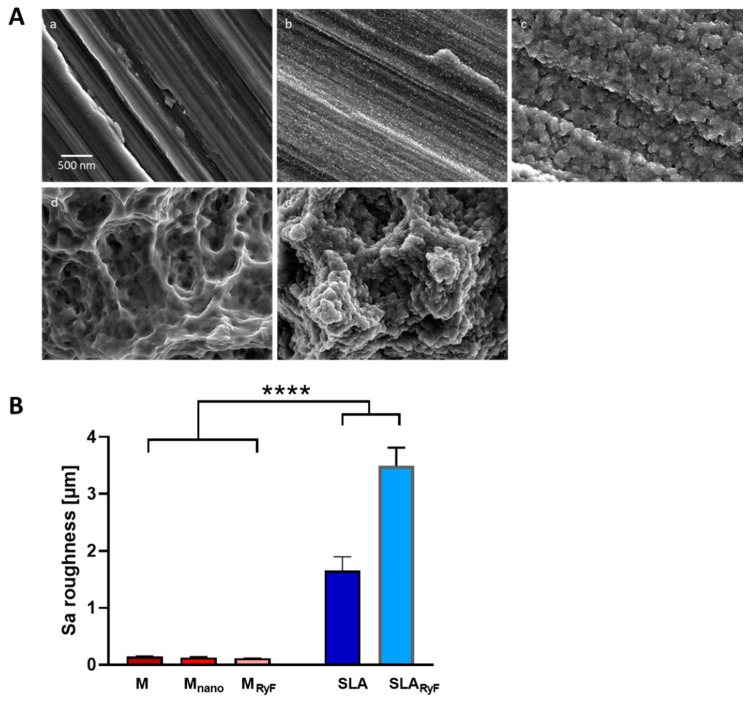
Scanning electron microscopy and roughness characterization of different surfaces. (**A**) Representative SEM images of the experimental titanium surfaces: (a) machined (M), (b) M_nano_, (c) M_RyF_, (d) SLA, and (e) SLA_RyF_. (**B**) Quantitation of the average surface roughness of the different surfaces (*n* = 5 independent samples per group analyzed via one-way ANOVA with Tukey`s post hoc test for multiple comparisons). Data are reported as mean ± SD (**** *p* < 0.0001).

For this study, five different modified titanium surfaces were selected (M, M_nano_, SLA, M_RyF_, and SLA_RyF_). On all three machined-based surfaces, parallel grinding marks are visible in the SEM micrographs, indicating typical anisotropy. The M surface (Figure 1A(a)) showed no additional nanostructures, while on the M_nano_ surface (Figure 1A(b)), clear nanostructures and fine spherical particles were visible. In the case of the M_RyF_ (Figure 1A(c)), a cauliflower-like secondary structure developed on the machined surface. The grinding grooves of the M surface were still clearly visible. The typical three-dimensional structure with different-sized pits, sharp ridges, and crevices formed by etching and sand blasting was seen on the SLA (Figure 1A(d)) surface. On the SLA_RyF_ (Figure 1A(e)) samples, the cauliflower-like structure already seen on the M_RyF_ was superimposed onto the original pits and crevices from the SLA base. The recorded EDX spectra show an additional fluorine peak in the spectrum for the R1234yF-fluorine-plasma-etched samples. While all the surfaces based on machined titanium were relatively smooth (Sa values shown in Figure 1B: M, 0.145 ± 0.015 µm; M_nano_, 0.131 ± 0.004 µm; M_RyF_, 0.116 ± 0.007 µm), those based on SLA appeared significantly rougher (SLA, 1.651 ± 0.245 µm; SLA_RyF_, 3.494 ± 0.321 µm) (*p* < 0.0001).

**Figure 2:**
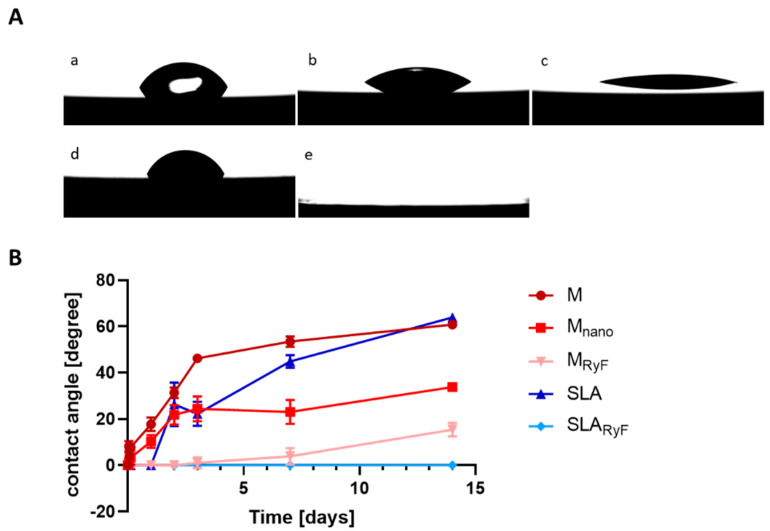
Contact angle measurement of the test samples at different time points: (**A**) Representative contact angle photos taken 14 days after O_2_ plasma cleaning: M (a), M_nano_ (b), M_RyF_ (c), SLA (d), and SLA_RyF_ (e). The SLA_RyF_ surface (e) was so hydrophilic that the water drop immediately spread over the surface and the initial contact with the surface could not be captured photographically. (**B**) Quantitative analysis of surface angles. Data are reported as mean ± SD; *n* = 3.

In order to investigate the wettability of the different surfaces, contact angle measurement was performed up to 14 days after cleaning with O_2_ plasma (Figure 2). Immediately after plasma treatment, all the surfaces tested had a contact angle of 0°, indicating initial superhydrophilic behavior. The machined surface displayed contact angles over 50° and already moderate hydrophilicity after only three days of storage. The contact angle reached 60.8° ± 1.8° (M) after 14 days, which is comparable with the wettability of the SLA surface (63.9° ± 0.9° after 14 days). According to our suggested wetting classification of the measured contact angles, both M and SLA were categorized as low wettable after 14 days. In contrast, the R1234yF plasma etching of both the M and SLA surfaces resulted in water contact angles below 20° for at least 14 days. M_RyF_, with contact angles of 15.4 ± 0.9°, was classified as strong hydrophilic and SLA_RyF_, with contact angles of even 0°, was classified as superhydrophilic (Figure 2B). The M_nano_ surface showed moderate hydrophilicity after 14 days, with contact angles of 33.8 ± 1.7°. Thus, a 14-day aging time led to a range of different hydrophilic titanium surfaces, from superhydrophilic to low-hydrophilic surfaces. None of the surfaces under investigation showed any hydrophobic wetting state after plasma cleaning or aging.

### 3.2. Fibroblast Attachment and Proliferation

Since soft-tissue attachment to the implants’ neck is as important as osseointegration for implantation success, the surfaces were also analyzed for the cellular response of human gingival fibroblasts (HGF).

**Figure 3:**
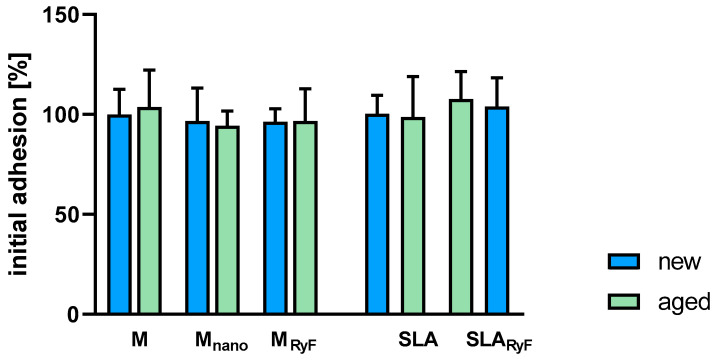
Initial adhesion of HGF cells was quantitatively determined with Alamar blue staining 1 h after seeding of the cells on the different titanium surfaces. The machined group (M new), after 24 h of cell incubation, was used as a control, set to 100%, and all other groups are referred to this. Three independent tests were performed with 4 discs per surface modification to be tested. The bar graph shows the mean ± SD.

**Figure 4:**
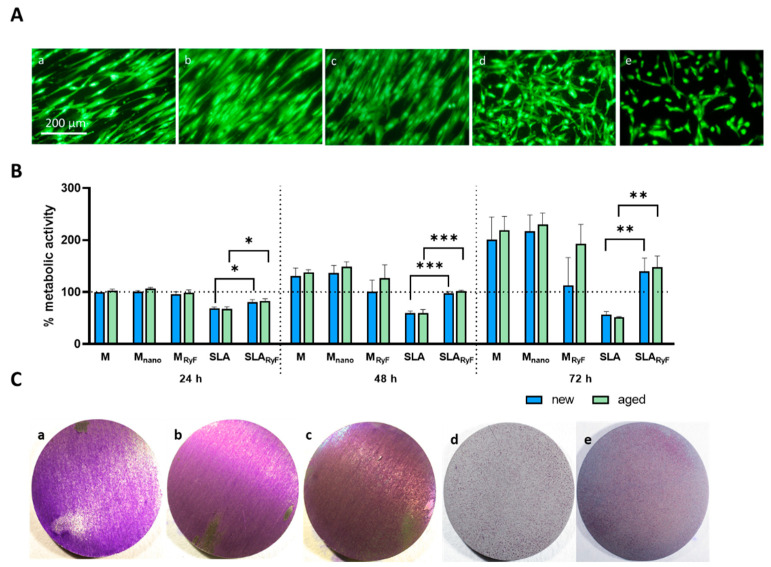
Evaluation of the cytocompatibility of different surfaces. (**A**) Representative fluorescent images of live/dead stained HGF cells seeded for 24 h on the different non-aged (new) sample discs (green = living; red = dead cells (not observed)). (**B**) Quantitative comparison of the metabolic activity of fibroblasts after 24 h, 48 h, and 72 h measured using CCK-8 assay. The machined group (M new), after 24 h of cell incubation, was used as a control and was set to 100%, and all other groups are displayed in relation to it. Three independent tests were performed, with 4 discs per surface modification to be tested. The bar graph shows the mean ± SD (* *p* < 0.05; ** *p* < 0.01; *** *p* < 0.001). (**C**) Representative images of crystal-violet-stained HGF fibroblasts growing on the non-aged sample discs for 72 h: (a) machined; (b) machined nano (M_nano_); (c) machined R1234yF (M_RyF_); (d) SLA; and (e) SLA R1234yF (SLA_RyF_).

To quantify the initial attachment of fibroblasts to different surfaces, Alamar blue staining was used to measure cell adhesion 1 h after seeding. It was observed that cells attached equally well to smooth and nanostructured surfaces. In addition, no significant differences were detected between the freshly O_2_-plasma-cleaned (new) and the 14-day-aged (aged) surfaces (Figure 3). As can be seen in Figure 4A, the fibroblasts tended to grow in a structured linear way following the parallel grinding marks of the surface when seeded on M, M_nano_, or M_RyF_, whereas they showed a more widespread morphology when cultivated for 24 h on the pits, sharp ridges, and crevices of the SLA and SLA_RyF_ surfaces.

The viability of the cells was monitored for a period of 72 h (Figure 4B). Even though the cells similarly attached to the different surfaces after 1 h of incubation, the cells growing on the SLA surfaces (SLA and SLA_RyF_) displayed lower metabolic activity compared to the M, M_nano_, or M_RyF_ surfaces after 24 h, 48 h, and 72 h of incubation. While viability remained low for the cells growing on SLA for 72 h, it increased over time for the cells growing on SLA_RyF_, reaching 70% (new) or 67% (aged) of the reference viability on the M surface after 72 h; meanwhile, SLA reached only 28% (new) and 24% (aged). A comparison of the viability of the cells on the SLA surface with the SLA_RyF_ showed significantly higher viability for the fluorine-plasma-etched surfaces on both the “new” and the “aged” surfaces (SLA “new” vs. SLA_RyF_ “new”: after 24 h, *p* = 0.0132; after 48 h, *p* = 0.0002; after 72 h, *p* = 0.0049) (SLA “aged” vs. SLA_RyF_ “aged”: after 24 h, *p* = 0.0118; after 48 h, *p* = 0.0006; after 72 h, *p* = 0.0016). However, no differences in cell viability were found between “new” and “aged” surfaces of the same surface modification in all the specimen groups.

The results of the metabolic activity measurement could also be visualized via crystal violet staining (Figure 4C), which showed that the lower metabolic activity of the cells on the rougher SLA surfaces was caused by the degree of occupancy, that is, the number of cells on the surface.

### 3.3. Osteoblast Differentiation

**Figure 5:**
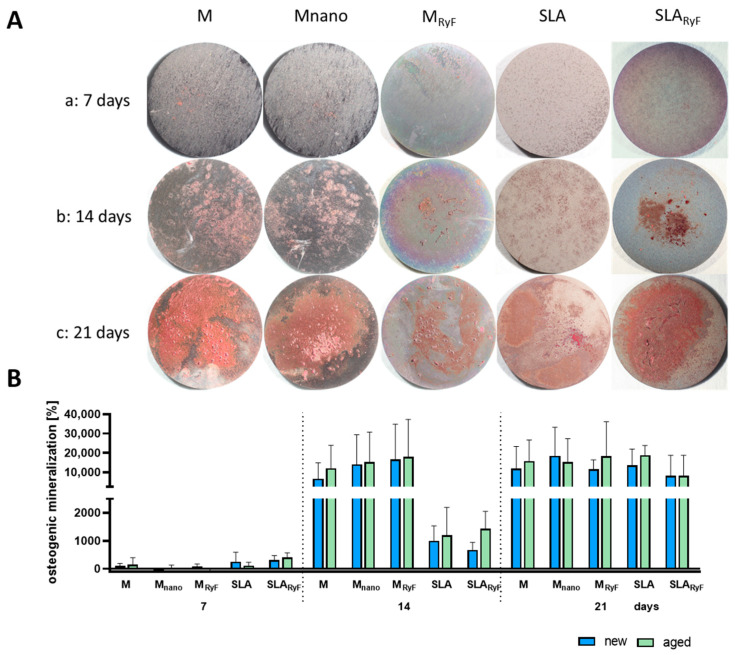
Osteogenic differentiation of osteoblasts on different “new” and “aged” titanium surfaces. (**A**) Representative images of the different “new” titanium discs with seeded SAOS-2 cells at different time points since the onset of differentiation. With prolonged differentiation, after (a) 7 days, (b) 14 days, and (c) 21 days, a visible increase in calcium phosphate formation stained with alizarin red was seen (disc diameter: 1.5 cm). (**B**) After photo documentation, the dye was dissolved from the cells and was quantified in a spectrophotometer. The machined “new” group, after 7 days, was used as a control and was set to 100%. The bar graph shows the mean ± SD (*n* = 3).

To investigate the osteogenic properties of the surface variants, the human-derived SAOS-2 osteoblast cell line was used. To test potential bone formation on the surfaces in vitro, SAOS-2 cells were stimulated to produce extracellular calcium phosphate deposits over 21 days. On the macroscopic imaging of the Alizarin-red-stained samples after 7, 14, or 21 days, there was no clear difference between the “new” surface (Figure 5A) and the “aged” surface (Figure S1), nor did the smooth surface and the nanostructured surface result in any substantial differences in calcium phosphate formation.

**Figure 6:**
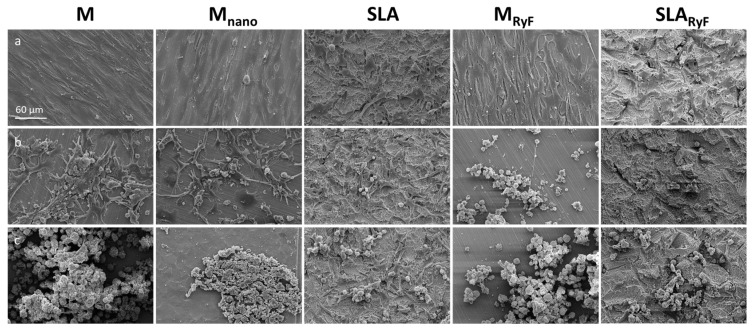
Representative scanning electron microscopy images showing the adhesion of SAOS-2 cells on the experimental non-aged titanium surfaces 7 days (**a**), 14 days (**b**), and 21 days (**c**) after the onset of osteogenic differentiation. With prolonged differentiation, morphological changes occurred, and calcium phosphate formation increased.

According to calcium quantification, mineralization occurred continuously on all the surfaces. After 14 days of differentiation, the cells on the M-based surfaces had deposited at least six times the amount of calcium phosphate than the cells on the SLA-based surfaces. This difference disappeared after 21 days since, at this time point, the quantity of mineral nodules was comparable between the SLA-based and the M-based surfaces, indicating similar calcium phosphate deposition in all the groups. Again, no differences between the “new” and the “aged” surfaces with the same modification could be detected (Figure 5B). The SEM images taken at the same timepoints as the calcium measurements show very clear morphological changes in the cells during the increasing calcium phosphate deposition (Figure 6). As with fibroblasts, SAOS-2 cells spread according to the surface structure.

## 4. Discussion

The physicochemical surface properties of an implant material are proven to influence the cellular response, thus influencing the long-term success of implantation [29,30,31,32]. Various surface modification techniques, like blasting, acid etching, or coating, have been developed in the last decades to modulate the physicochemical and biological properties of titanium implants [33,34]. Titanium implants’ topographical micro-/nanofeatures, in combination with their wettability, have been shown to modulate biological responses in in vitro and animal studies [16,35,36,37]. Highly structured implant surfaces are assumed to increase the implant–bone surface contact area, thus promoting the biological fixation [38]. Hence, the micro- and nanoscaled structuring of implant surfaces aims to enhance cell attachment and tissue healing, which are critical steps in osseointegration [35]. However, while the role of surface topography in bioresponses has received extensive attention, only a few studies have explored the relationship between implant wettability and cellular response [10,35,38].

This study focused on investigating two physical and chemical modification strategies that may impact implant healing. Firstly, the commercially available surfaces (machined and SLA) were additionally plasma-etched with fluorine gas (R1234yF) to create an additional and novel nanoroughness on the surfaces, as can be seen on the SEM images. Besides topography and roughness, the surface wettability or hydrophilicity of implants can also play a role in osseointegration. All the surfaces tested in our study were freshly O_2_-plasma-cleaned (new) and were compared to the “aged” surfaces (14 days after plasma cleaning) to further investigate how hydrophilicity affects cell attachment, viability, and osteoblast differentiation.

It was shown that the O_2_-plasma-cleaned surfaces had a water contact angle of 0° and were thus superhydrophilic. During storage in the ambient atmosphere (aging), the initial hydrophilicity of the material decreased over time. The superhydrophilicity of the fluorine-gas-etched surfaces, however, was maintained over a longer period of time. Several in vitro and in vivo studies showed that increased hydrophilicity is a crucial factor for fast implant healing since initial protein adhesion is promoted. It is well known that hydrophilicity is the main driving force in protein folding [39,40].

Hydrophilic surfaces maintain protein conformation and function, while hydrophobic surfaces seem to induce denaturation [41]. As a critical factor determining cell attachment and proliferation, rapid protein attachment should provide an advantage and can influence tissue–implant integration. In this study, we did not find significant differences between the hydrophilic and “aged” surfaces. Neither the initial attachment nor the viability of the HGF cells over 72 h showed a significant difference. There was also no difference in SAOS-2 osteoblast differentiation over 21 days between the “new” and “aged” surfaces. Despite higher mineralization on the M-based surfaces after 14 days (increased by a factor of 12,360 for M “new” within 7 days) compared to the SLA-based surfaces (SLA “new” increased by a factor of 394, where SLA_RyF_ “new” increased by 216), the values were comparable after 21 days in all the groups, indicating no profound impact neither of the different surface topographies nor of different wettability on osteoblast differentiation. Some published long-term in vivo studies on animals also showed no significant positive effect of hydrophilized implant surfaces [42]. On the other hand, a number of in vivo studies have demonstrated that hydrophilicity enhances early osseointegration [9,42,43].

It has to be considered that, even though aging lowered the superhydrophilic state of all the surfaces except plasma-etched SLA, none of the investigated surfaces were hydrophobic after aging and at the time point of biological testing. In this respect, our study did not compare real hydrophobic surfaces with superhydrophilic ones but different surface types, each with individual lowered hydrophilicity after aging.

Taken together, our results as well as findings from other groups suggest that hydrophilicity may not be the only determining factor regarding cell attachment and osseointegration. Other factors may play a decisive role in biological responses. These include surface charge, chemical composition, oxidation state, carbonate levels, and hydrocarbon contaminants.

Nanostructures, along with microstructures, accelerate osseointegration and reduce the healing time by improving adhesion and the proliferation time. In search of new, improved surfaces, this study investigated the plasma etching of titanium surfaces with the fluorine gas 2,3,3,3-tetrafluoropropene (R1234yF), which resulted in an additional nanostructure on the sample surfaces. Subsequently, it led to sustained superhydrophilicity and to fluorine accumulation on the surface.

Our study indicates that the plasma etching, with 2,3,3,3-tetrafluoropropene, of the machined and SLA surfaces did not have an adverse effect on the biological performance of the osteoblast cell line SAOS-2. In the osteoblasts, neither the additional nanostructure on the surfaces nor the fluorine content affected the differentiation or performance of the cells. These results agree with Pham et al.’s study [44], who examined the influence of fluoride on the proliferation and differentiation of primary human osteoblasts on HF-etched, untreated TiO_2_ and Ti surfaces.

Moreover, there were no differences in cell differentiation over 21 days in this study as well. This contrasts with the results of Lamolle et al. [45]. When comparing titanium discs etched with increasing HF etching times with those not etched, it was shown that surfaces with a higher fluoride content had a less cytotoxic effect and that more cells were present on the surfaces. However, they used the mouse osteoblast cell line MC3T3-E1 for this study rather than human cells, which could explain the different findings. It is also difficult to conclude whether the observed differences are due to the fluoride content of the surfaces, the increased hydrophilicity, or the enlarged roughness caused by etching. However, these contradictory results could also be due to the fact that fluorine plasma etching, rather than wet chemical HF etching, was carried out in this study.

Other studies hypothesize that fluorine-containing surfaces propagate the host-to-implant reaction in early osseointegration [43,46,47], which we could not confirm for the SAOS-2 cell line. In the fibroblast cell line HGF, however, higher viability was detected on the fluorine-plasma-etched samples after 24, 48, and 72 h (SLA_RyF_ “new”: after 24 h, 1.18 × increase; after 48 h, 1.64 × increase; after 72 h, 2.85 × increase) (SLA_RyF_ “aged”: after 24 h, 1.22 × increase; after 48 h, 1.71 × increase; after 72 h, 2.85 × increase). This contradictory result could be due to the additional nanostructure or to the fluorine content on the fluorine-plasma-etched sample surfaces to which osteoblasts react differently than fibroblasts.

Overall, which surface characteristics are the key players influencing the cascade of biological reactions towards osseointegration and soft-tissue sealing is still controversially discussed. Next to wettability and roughness, it is pivotal to analyze the influence of further physiochemical aspects, like surface tension or surface energy [48].

## 5. Conclusions

In this study, the biological responses to different wettability levels and nanoscaled structures, which were superimposed, e.g., via plasma fluorine etching, on experimental machined (M) and combined blasted and acid-etched (SLA) titanium implant surfaces were examined. These surface-modifying processes resulted in different surface roughness values. All the surfaces were superhydrophilic directly after a plasma-cleaning process, whereas the hydrophilicity moderately decreased again with aging on the M, M_nano_, and SLA surfaces. In contrast, the surfaces etched with fluorine gas (M_RyF_ and SLA_RyF_) showed a long-term, strong hydrophilicity that lasted for at least 14 days.

Although different in surface roughness and hydrophilicity, the initial adhesion of fibroblasts was comparable on all the surfaces. However, after 72 h, the cell coverage rate of fibroblasts and their viability were clearly reduced on the SLA surface. Interestingly, this decrease was not observed on fluorine-gas-etched SLA, indicating that the SLA_RyF_ surface shows better biocompatibility for fibroblasts.

Calcium phosphate deposition by osteoblasts on all the surfaces did not show any significant differences after 21 days. We therefore conclude that other factors are likely to be decisive for cell attachment, cell viability, and osseointegration in addition to surface roughness and wettability. Surface energy and fluorine compounds are two factors that might play a role. Considering the lack of further characterization in terms of surface energy, charge, and contamination or the fact that every surface modification changes several parameters, it seems very difficult to systematically modify a single parameter as a whole. In order to determine the main factors influencing the observed cell reaction or bacterial adhesion and colonization, future analysis will need to be performed.

## Figures and Tables

**Table 1:** Overview of specimen groups evaluated in the present study.

Group	Surface Modification
M	Machined surface without further surface treatment
M_nano_	Plasma cleaning followed by hydrothermal treatment with sodium chloride
M_RyF_	Plasma etching of machined surface with 2,3,3,3-tetrafluoropropene
SLA	Blasted with large grits of 0.25–0.50 mm corundum and acid-etched in a mixture of HCl and H_2_SO_4_.
SLA_RyF_	Plasma etching of SLA surface with 2,3,3,3-tetrafluoropropene

## Data Availability

Data are contained within the article.

## Supplementary Materials

The following supporting information can be downloaded at: https://www.mdpi.com/article/10.3390/ma16237307/s1, Figure S1: Osteogenic differentiation of osteoblasts on different hydrophobic titanium surfaces. Representative images of the different hydrophobic titanium disks with seeded SAOS-2 at different time points since onset of differentiation. With prolonged differentiation after (a) 7 days, (b) 14 days and (c) 21 days a visible increase in calcium phosphate formation stained with alizarin red is seen (disks diameter: 1.5 cm).
