# Supplementary material for: Biological Performance of Titanium Surfaces with Different Hydrophilic and Nanotopographical Features
Source: Materials (Basel). 2023 Nov 24;16(23):7307. doi: 10.3390/ma16237307 (PMC10707616; doi:10.3390/ma16237307)
Supplement: Supplementary file 1 [file materials-16-07307-s001.zip › materials-2727119-supplementary.pdf]

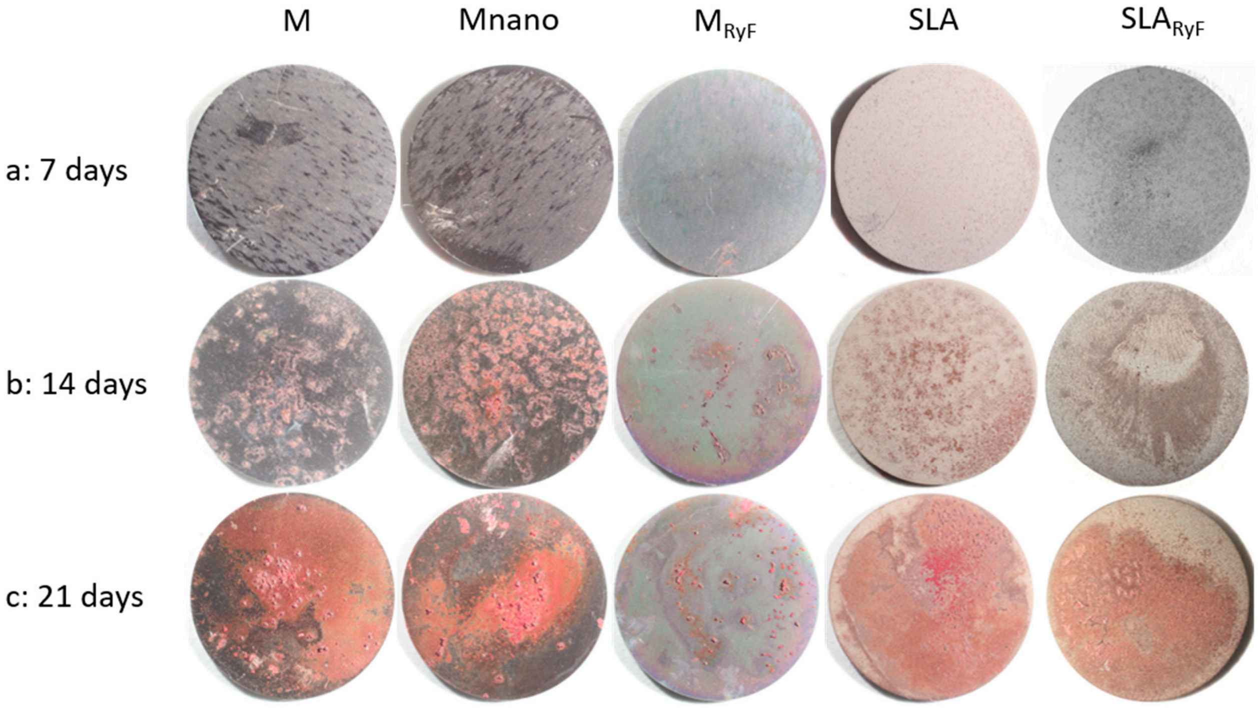

Figure S1. Osteogenic differentiation of osteoblasts on different hydrophobic titanium surfaces. Representative images of the different hydrophobic titanium disks with seeded SAOS-2 at different time points since onset of differentiation. With prolonged differentiation after (a) 7 days, (b) 14 days and (c) 21 days a visible increase in calcium phosphate formation stained with alizarin red is seen (disks diameter: 1,5 cm).
